# Statistics of eye movements in scene categorization and scene memorization

**DOI:** 10.1186/1471-2202-13-S1-P8

**Published:** 2012-07-16

**Authors:** Xin Chen, Weibing Wan, Zhiyong Yang

**Affiliations:** 1 Brain & Behavior Discovery Institute, Georgia Health Sciences University, Augusta, GA, 30912, USA; 2 Department of Ophthalmology, Georgia Health Sciences University, Augusta, GA, 30912, USA; 3 Vision Discovery Institute, Georgia Health Sciences University, Augusta, GA, 30912, USA

## 

Humans can grasp the gist of complex natural scenes in less than 100 milliseconds. To model this rapid scene perception, we developed ultra-sparse structural representations of natural scenes. In this model, a natural scene is represented by a probability distribution based on a set of natural scene structures and their spatial concatenations. In contrast to bottom-up, image-based processing and recent scene gist models based on global features, the structural representations in our model require no isolation of objects, nor computation of global scene features. We tested this model by comparing the patterns of eye movements of human subjects while they were performing two scene-viewing tasks, scene categorization and scene memorization[1,2]. Since human vision system always actively seeks visual information needed to support current cognitive requirements[3], we predicted that the subjects would scan more informative patches of the scenes in scene categorization than in scene memorization.

We examined the statistics of the eye movements of the subjects in the two tasks. We found that: 1) the average number of fixations in scene categorization was significantly less than that in scene memorization (t = 7.02, p < 0.001); 2) the average fixation duration in scene categorization was significant longer than that in scene memorization (t = 2, p = 0.05); 3) the subjects executed more long fixations in scene categorization than in scene memorization; and 4) the total length of the scan paths in scene categorization was shorter than that in scene memorization (t = 5.48, p < 0.01). We also developed models of scene categorization and scene memorization using the scene patches sampled at the fixations and found that the subjects extracted more informative scene patches in scene categorization than in scene memorization.

Therefore, the subjects tended to coarsely scan the whole scenes when performing scene memorization, but opted to scan smaller scene patches with more detailed information when performing scene categorization. This observation supports our model of visual scenes as spatial concatenations of natural scene structures, each of which conveys a variety of amount of information about scene identity and category.
